# Variants of Genes Involved in Metabolism of Folate Among Patients with Breast Cancer: Association of *TYMS* 3R Allele with Susceptibility to Breast Cancer and Metastasis

**DOI:** 10.30699/ijp.2020.117676.2283

**Published:** 2020-11-10

**Authors:** Zohreh Rahimi, Maryam Bozorgi Zarini, Ziba Rahimi, Ebrahim Shakiba, Asad Vaisi-Raygani, Mohammad Taher Moradi, Kheirolah Yari

**Affiliations:** 1 *Medical Biology Research Center, Kermanshah University of Medical Sciences, Kermanshah, Iran*; 2 *Department of Clinical Biochemistry, Medical School, Kermanshah University of Medical Sciences, Kermanshah, Iran*; 3 *Fertility and Infertility Research Center, Kermanshah University of Medical Sciences, Kermanshah, Iran*

**Keywords:** Breast cancer, Thymidylate synthase, Methionine synthase, Methionine synthase reductase, Polymorphism, Metastasis

## Abstract

**Background & Objective:**

Breast cancer (BC) is known to be the most prevalent cancer among women. One-carbon metabolism disturbance might play an important role in the etiology of BC. The present study aimed to investigate the thymidylate synthase (TYMS), 5-methyltetrahydrofolate-homocysteine methyltransferase (MTR), and methionine synthase reductase (MTRR) variants as good candidates for studying the role of genetic variants of folate metabolizing enzymes in the risk of BC.

**Methods:**

The present case-control study includes 100 BC patients and 141 healthy females. The *TYMS* 2R/3R (rs34743033), *MTR* c.2756A>G (rs1805087), and *MTRR* c.66A>G (rs1801394) variants were detected by polymerase chain reaction (PCR), PCR-restriction fragment length polymorphism (RFLP), and a designed amplification-refractory mutation system (ARMS) method, respectively.

**Results:**

The 3R allele of *TYMS* enhanced the risk of BC by 2.84-fold (*P*<0.001). In the presence of *TYMS* 3R/3R, compared to *TYMS* 2R/3R, there was a trend toward enhancing the risk of metastasis by 4.15-fold (95% CI: 0.96-17.85, *P*=0.055). The frequencies of *MTR* c.2756A>G and *MTRR* c.66A>G variants were not significantly different among patients and controls.

**Conclusion:**

We observed that the *TYMS* 3R is a risk allele for susceptibility to BC and this allele may increase the risk of metastasis in BC patients. .

## Introduction

Breast cancer (BC) is the most prevalent cancer among women accounting for 16% of all cancers in females (1). The increase rate of BC incidence is 3-4% in developing countries (2), in which BC occurs around one decade earlier compared to developed countries. Early diagnosis of BC is critical for treatment of the disease and reducing the mortality rates (3). 

Genetic alterations in a multistep process result in normal epithelial cells transformation of mammary into highly malignant cells (4). Population studies suggest a role for gene variants and the mutations as the strong risk factors affecting the individual differences in the BC susceptibility (5).

Since one-carbon metabolism (OCM) has a major role in the DNA synthesis and methylation and interaction between genetic and epigenetic processes, OCM disturbance might have an important role in the etiology of BC (6). The pathway of OCM plays a critical role in the integrity of genome, the gene expression, and the methylation of DNA. So, dysregulation of the pathway might be involved in the risk of BC and its severity (7). Single nucleotide polymorphisms (SNPs) in folate-related genes are suitable candidates for investigating the role of these gene variants in BC risk (8). The thymidylate synthase (TYMS), 5-methyltetrahydrofolate-homocysteine methyltransferase (MTR), and methionine synthase reductase (MTRR) are suitable genes to study the role of variants of genes involved in the metabolism of folate in the BC risk.

The 5,10-methylene tetra hydrofolate (THF) is the common substrate for two key enzymes of TYMS and methylenetetrahydrofolate reductase (MTHFR). These enzymes catalyze the conversion of deoxyuridine monophosphate (dUMP) to deoxythymidine monophosphate (dTMP) and flavin adenine dinucleotide (FAD)-dependent reduction of 5,10-methylene THF to 5-methyl THF, respectively (9). The dTMP is necessary for DNA synthesis and repair. This reaction plays an important role in intracellular de novo synthesis of thymidylate (10). 

The TYMS gene locates at 18 p11.32. The important target for the chemotherapy drugs such as 5-fluorouracil (5-FU) is TYMS (11). The most common variation in TYMS (18p11.32) is a double (2R) or triple (3R) 28-bp repeat sequence (rs34743033) in the 5'-untranslated region of the promoter enhancer that affects the protein expression in cancer cells (12). In the presence of the *TYMS* 3R, compared with 2R, there is 2.6-fold more TYMS expression and enhancing the TYMS enzymatic activity.

The MTR gene encodes an enzyme which requires vitamin B12 and catalyzes the remethylation of homocysteine to methionine using 5-methyl THF (12). The MTR gene locates at 1q43 (13). The common polymorphism of *MTR* c.2756A>G (rs1805087) reduces the enzyme activity due to the conversion of aspartate to glycine in the protein, and induces the increase of homocysteine and DNA hypomethylation (12). This polymorphism has been suggested as a good candidate for predisposition to BC risk (13-15). 

MTRR enzyme helps in the regeneration of functional MTR by reductive methylation and maintains the active form of MTR (16). The gene of MTRR locates at 5p15.31. The polymorphism of *MTRR* c.66A>G (rs1801394) results in replacement of isoleucine with methionine at amino acid 22 in the protein (17). The presence of this polymorphism leads to lower affinity of the variant enzyme for methionine synthase (16).

The aim of preset study was to investigate the role of *TYMS* 2R/3R (rs34743033), *MTR* c.2756A>G (rs1805087), and *MTRR* c.66A>G (rs1801394) gene variants in susceptibility to BC among a population with Kurdish ethnic background from Western Iran.

##  Materials and Methods

**Sample**

We studied 100 BC patients (99 females and 1 male) aged 49.5±10.2 years (29–79 years) and 141 healthy females aged 38.7±9.4 years (range 28–70 years). Diagnosis of BC was based on standard clinical, radiological, and histological parameters. All patients had been admitted to a university hospital affiliated to the Kermanshah University of Medical Sciences (KUMS), Iran. The ethnic background of both patients and controls was Kurdish. The Ethics Committee of KUMS approved the study. All patients and healthy controls agreed to participate in the study and a written informed consent was obtained from all participants prior to conducting the study. The study was in accordance with the principles of the Helsinki II declaration. 

**Genotyping**

From each participant, 5 ml of whole blood with anticoagulant of Ethylenediaminetetraacetic acid (EDTA) was taken. Using treatment of whole blood with proteinase K and subsequent phenol–chloroform extraction and ethanol precipitation, genomic DNA was isolated from leukocytes (18). 

The concentration and purity of DNA were determined by a Nanodrop spectrophotometer (Thermo Fisher Scientific, Waltham, Massachusetts, USA). 

**Thymidylate Synthase**

**Fig 1:**
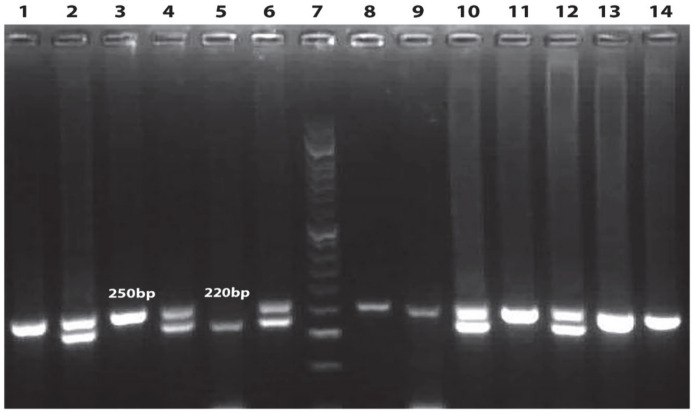
Electrophoresis of *TYMS* PCR products was done using agarose gel. From left to right columns 1, 3, 8, 9, 11, 13, 14 indicate the genotype of 3R3R, columns 2, 4, 6, 10, 12 demonstrate the genotype of 2R3R and column 5 shows the genotype of 2R2R. Column 7 demonstrates DNA molecular marker

The genomic region of *TYMS* was amplified by polymerase chain reactions (PCR) using the forward primer 5´GTG GCT CCTGCG TTT CCC CC 3´and the reverse primer 5´CCA AGCTTG GCT CCG AGC CGG CCA CAG GCA TGG CGCGG 3´to detect the tandem repeat sequences in the 5´-terminal of the *TYMS* regulatory region (*TYMS* 2R/3R (rs34743033). The parameters of PCR thermal cycling were: 1 cycle at 94^o^C for 5 min, 40 cycles by 94^o^C for 60s, 63^o^C for 60s, and 72^o^C for 60s followed by final extension at 72^o^C for 10 min. In the presence of homozygotes of double repeat (*TYMS* 2R2R) a 220-bp fragment is produced, while heterozygotes (*TYMS* 2R3R) produce two fragments of 220- and 250-bp. In the presence of homozygous *TYMS* triple repeat (*TYMS *3R3R) a fragment with 250-bp is produced (Figure 1) (12).

***MTR*** **c.2756A>G**

The *MTR* c.2756A>G polymorphism was identified using the forward primer 5´TGT TCCAGA CAG TTA GAT GAA AAT C 3´and 5´GAT CCA AAG CCT TTT ACA CTC CTC 3´as reverse primer. The parameters of PCR thermal cycling were: 1 cycle at 94^o^C for 5 min, 40 cycles by 94^o^C for 60s, 53^o^C for 60s, and 72^o^C for 60s followed by final extension at 72^o^C for 10 min. The 211-bp PCR product (10 to 15 µl) was digested using five units of the Hae III restriction enzyme at 37ºC overnight. The presence of *MTR* c.2756A>G polymorphism creates a recognition sequence for Hae III, which digests the PCR product with 211-bp into 131- and 80-bp fragments (12).

***MTRR*** **c.66A>G**

**Fig. 2.**
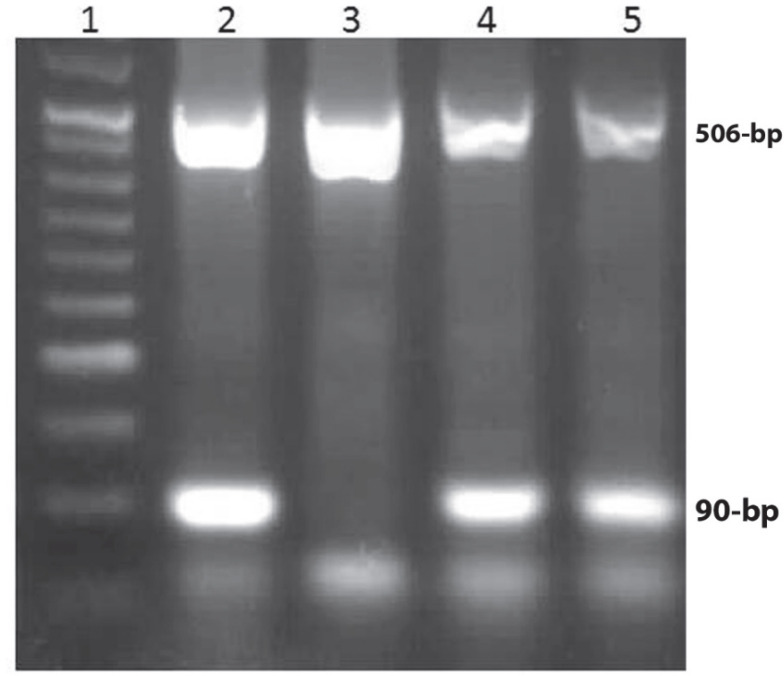
The pattern of PCR-RFLP products of the *MTRR* c.66A>G on agarose gel electrophoresis was obtained. Both lanes 2 and 3 (wild and mutant alleles from the same sample) indicate homozygous *MTRR* AA genotype. Lanes 4 and 5 (wild and mutant alleles from the same sample) demonstrate *MTRR* AG genotype. Lane 1 depicts a 50-bp molecular weight

The genotypes of *MTRR* c.66A>G was detected by a designed amplification-refractory mutation system (ARMS) method. We designed primers with Oligo7 software that consisted of a primer for wild allele as 5'- TGTACCACAGCTTGCTCACTT-3', a primer for mutant allele with the sequence of 5'- TGTACCACAGCTTGCTCACTC-3', and a common primer of 5'- TGAAGTGATGAGGAGGTTTC-3'. The parameters of thermal cycler for gene amplification were: 1 cycle at 94°C for 5 min; 35 cycles: denaturation (94°C for 35 s), annealing (58°C for 35 s), extension (72°C for 45 s), and final extension (72°C for 5 min). The PCR product of wild and/or mutant allele was a fragment with 90-bp. A fragment with 506-bp from prothrombin gene was amplified using the primers 5' GCA CAG ACG GCT GTT CTC TT 3' and 5' ATA GCA CTG GGA GCA TTG AAG C 3', and was used as internal standard (19) (Figure 2).

**Statistics**

Using allele frequency calculation method, the frequency of the alleles was calculated. Difference in the alleles and genotypes frequency between both groups and its significance were tested using the χ^2 ^method. Data on quantitative characteristics are explained as mean ± standard deviation (SD). Odds ratios (OR), that estimates the relative risk of disease, and 95% confidence interval (CI) were detected using the SPSS software logistic regression. Quantitative data were compared using Student’s t test. The level of P-value<0.05 was used for the statistical significance. The SPSS version 16 was used for statistical analyses (SPSS Inc., Chicago, IL., USA).

## Results

Among participants, 39 individuals (39%) had a family history of cancer among first degree relatives. Based on the available files from 66 patients, there were 51 patients (77.3%) with lymph node metastasis. Also, there were 13 patients (19.7%) with tumor stage I, 42 patients (63.6%) with tumor stage II, and 11 patients (16.7%) with tumor stage III. Further, based on available data from files of 82 patients, the histological type of tumor was as 73 (89%) with invasive ductal carcinoma, 8 (9.8%) with invasive lobular carcinoma, and 1 (1.2%) with in situ type.

The genotypes and alleles frequency of *TYMS* 2R/3R in patients with BC and controls are indicated in Table 1. *TYMS* 28-bp repeat 2R2R, as a reference genotype, was identified in 9 BC patients (9%), 2R3R was observed in 38 patients (38%), and 3R3R was identified in 53 patients (53%). The frequencies of 2R2R, 2R3R, and 3R3R genotypes in 141 healthy individuals were 20.6, 63.8, and 15.6%, respectively. The frequency of 2R allele among patients and healthy individuals were 28 and 52.5%, respectively (Table 1). The frequency of 3R allele was 72% in patients and 47.5% in controls (*P*<0.001). Higher frequency of 3R allele in patients enhanced the BC risk (OR=2.84, 95% CI: 1.92–4.18, *P*<0.001) (Table 1).

**Table 1:** Distribution of *TYMS* 2R/3R polymorphism in breast cancer patients and healthy individuals

*TYMS*	Patients n= 100 n (%)	Controls n=141 n (%)	χ^2^, p	OR (95% CI), *P*
2R 2R	9 (9)	29 (20.6)	-	-
2R 3R	38 (38)	90 (63.8)	32.1, <0.001	0.175 (0.094-0.32), <0.001
3R 3R	53 (53)	22 (15.6)	22.5, <0.001	2.78 (1.77-4.36), <0.001
Allele				
2R	56 (28)	148 (52.5)	28.7, <0.001	2.84 (1.92- 4.18), <0.001
3R	144 (72)	134 (47.5)		

Overall χ^2^=38.6, P-value<0.001comparing three genotypes

In BC patients with metastasis, a significantly higher frequency of *TYMS* 3R3R (n=28, 60.9%) was detected compared to 2R3R (n=18, 39.1%). The presence of *TYMS* 3R/3R, compared to 2R/3R, tended to elevate the risk of metastasis by 4.15-fold (95% CI: 0.96-17.85, *P*=0.055)

Distribution of *MTR* 2756A>G genotypes and alleles in patients and controls are depicted in Table 2. The common genotype of *MTR* AA was found in 59 patients (60.2%), the AG genotype in 35 patients (35.7%) and the homozygous genotype of GG were identified in 4 patients (4.1%). In healthy controls, 75 subjects (53.2%) had the AA genotype, 52 subjects (36.9%) had the AG genotype, and 14 subjects (9.9%) had the GG genotype. The frequency of G allele was 21.9% in patients and 28.4% in controls (*P*=0.1) (Table 2). 

**Table 2:** Comparison of *MTR* c.2756A>G genotypes and alleles between patients and control group

*MTR* c.2756A>G	Patients n= 98 n (%)	Controls n=141 n (%)	χ^2^, *P*
Genotype			
AA	59 (60.2)	75 (53.2 )	
AG	35 (35.7)	52 (36.9)	0.36, 0.54
GG	4 (4.1)	14 (9.9)	3.19 (0.074)
Allele			
A	153 (78.1)	202 (71.6)	2.64, 0.1
G	43 (21.9)	80 (28.4)	

Overall χ^2^=3.25, 0.19 comparing three genotypes

The frequency of genotypes and alleles of *MTRR* 66A>G in patients and healthy individuals are depicted in Table 3. The frequency of *MTRR* GG genotype was 16.1% in patients compared to 13.5% in controls. The frequency of *MTRR* G allele was not significantly different among BC patients and controls (49% vs. 51.4%, *P*=0.65) (Table 3).

**Table 3:** The frequency of *MTRR* c.66A>G polymorphism in breast cancer patients compared to control group

*MTRR* c.66A>G	Patients n= 99 n (%)	Controls n=141 n (%)	χ^2^, *P*
Genotype			
AA	18 (18.2)	15 (10.6)	-
AG	65 (65.7)	107 (75.9)	2.88, 0.09
GG	16 (16.1)	19 (13.5)	0.36, 0.54
Allele			
A	101 (51)	137 (48.6)	0.19, 0.65
G	97 (49)	145 (51.4)	

Overall χ^2^= 3.28, P-value=0.19

## Discussion

TYMS is a necessary enzyme for biosynthesis of DNA. In the presence of three copies of tandem repeats (*TYMS* 3R), compared with two copies of tandem repeats (2R), there is higher TYMS expression enhancing the TYMS enzymatic activity (20).

In the present study we detected a significantly increased risk of BC by 2.84-fold in the presence of *TYMS* 3R. A better response to 5-FU therapy and increased overall survival has been observed in patients with the genotype of *TYMS* 2R/2R or *TYMS* 2R/3R compared to *TYMS* 3R/3R (20). The role of ethnicity in different frequency of gene's variants involved in the metabolism of folate and methionine has been indicated (12). In Mexican females, *TYMS* 2R/3R was not related to the risk of BC (21). Also, in three studies from Asia, Japan, China, and India, *TYMS *polymorphism did not influence the risk of BC (22-24). A meta-analysis indicated that *TYMS* 2R/3R might increase the risk of BC in Caucasian females and suggested the role of ethnic background for *TYMS* variants in BC susceptibility (25).

There was no significant difference in the distribution of *MTR* 2756A>G variants between BC patients and healthy individuals. The role of this polymorphism in the BC risk is controversial. Among southeastern Asians, three studies from china found a significant association between *MTR* 2756A>G variants and BC risk (26-28). However, in reports from China, Japan, and India, no association was detected between *MTR* 2756A>G polymorphism with the risk of BC (22, 24, 29). In a meta-analysis, this polymorphism was not correlated with the BC risk considering all populations. However, subgroup analysis indicated that the 2756G allele was correlated with a reduced risk in Caucasians (30). The reasons for various results obtained in Asians (the lack of association between this polymorphism with the risk of BC) and in Europeans (decreased risk of BC in the presence of G allele) could be attributed to genetic background diversity (ethnicity) and gene-environment interactions. Other factors that might explain the heterogeneity of results among the existing studies include: the differences in sample size, the different genetic background of studied populations, the differences in study design and conduct, the interaction between gene–gene and gene–environment, and the absence of stratification of data according to folate intake (30).

In our study, we observed the lack of association between *MTRR* 66A>G polymorphism with the risk of BC. The *MTRR* 66A>G variants were not associated with the risk of BC among Indian, American, Canadian, and Syrian females (24, 31-33). The Shanghai Breast Cancer Study among Chinese female patients reported the absence of a significant association between the risk of BC and *MTR *2756A>G or *MTRR* 66A>G genotypes (34). In a meta-analysis conducted by Hu *et al.* (16) considering 7,097 cases and 7,710 controls, the *MTRR* 66A>G variants were not associated with BC risk. But among Chinese female patients with BC, MTRR had a protective role and decreased the risk of BC (35). Also, in a meta-analysis by Naushad *et al.* (9) which consisted of 62 case-control studies from 17 different populations including 18,117 BC cases and 23,573 healthy controls, the *MTR* 2756A>G indicated a borderline protective role against BC (OR=0.78, 95% CI: 0.75–0.82). In silico analysis revealed that the *MTR *2756A>G could induce benign damage to the MTR protein while the *MTRR* 66A>G indicated a deleterious effect on MTRR protein. Regarding the action of *MTR* and *MTRR* together in a 1:1 stoichiometric ratio to produce the holoenzyme complex, it seems the variant alleles of *MTR* and *MTRR* act in synergy in modulation of BC risk (34).

## Conclusion

The results of the present study indicates that the *TYMS* 3R allele may be a risk factor for BC development in our population. Also, in the presence of *TYMS* 3R/3R, a tendency and increased risk for BC metastasis may exist. However, *MTR* 2756A>G and *MTRR* 66A>G variants are not significantly associated with the risk of BC development. In addition, there is no significant association between these genetic variants with the histopathological characteristics of the patients.
